# Thyroid Dysfunction and COVID-19: The Emerging Role of Selenium in This Intermingled Relationship

**DOI:** 10.3390/ijerph19116912

**Published:** 2022-06-05

**Authors:** Francesca Gorini, Laura Sabatino, Alessio Coi, Giorgio Iervasi, Cristina Vassalle

**Affiliations:** 1 Institute of Clinical Physiology, National Research Council, 56124 Pisa, Italy; laura.sabatino@ifc.cnr.it (L.S.); alessio.coi@ifc.cnr.it (A.C.); iervasi@ifc.cnr.it (G.I.); 2 Fondazione CNR-Regione Toscana Gabriele Monasterio, 56124 Pisa, Italy

**Keywords:** COVID-19, coronavirus, thyroid disease, thyroiditis, selenium

## Abstract

COVID-19 represents a worldwide public health emergency, and, beyond the respiratory symptoms characterizing the classic viral disease, growing evidence has highlighted a possible reciprocal relationship between SARS-CoV-2 infection and thyroid dysfunction. The updated data discussed in this review suggests a role of SARS-CoV-2 infection on the thyroid gland, with multiple thyroid pictures described. Conversely, no conclusion can be drawn on the association between pre-existing thyroid disease and increased risk of SARS-CoV-2 infection. In this scenario, selenium (Se), an essential trace element critical for thyroid function and known as an effective agent against viral infections, is emerging as a potential novel therapeutic option for the treatment of COVID-19. Large multicentre cohort studies are required to elucidate the mechanisms underlying thyroid dysfunction during or following recovery from COVID-19, including Se status. Meanwhile, clinical trials should be performed to evaluate whether adequate intake of Se can help address COVID-19 in Se-deficient patients, also avoiding thyroid complications that can contribute to worsening outcomes during infection.

## 1. Introduction

The outbreak of coronavirus disease19 (COVID-19) rapidly disseminated globally after the first Chinese case in late December 2019, becoming a public health emergency for the entire world, often overwhelming healthcare systems [1].

Severe acute respiratory syndrome coronavirus 2 (SARS-CoV-2) is a highly transmissible and pathogenic coronavirus which, as a novel betacoronavirus, shares 79% genome sequence identity with severe acute respiratory syndrome coronavirus (SARS-CoV) and 50% with Middle East respiratory syndrome coronavirus [2]. This RNA virus is responsible for an acute respiratory illness with varying degrees of severity, and patients with pre-existing comorbidities are at a greater risk of COVID-19 occurrence with more adverse clinical outcomes [3]. Beyond classic respiratory symptoms, COVID-19 represents a systemic disease acting through different pathological mechanisms (e.g., inflammation, hypercoagulability, and alterations of the renin-angiotensin-aldosterone system), because of which some patients with SARS-CoV-2 infection may show a range of complications in different extrapulmonary organs [4]. Among these extrapulmonary manifestations, adverse effects involving the endocrine system have been observed at multiple levels [4]. In particular, a growing body of evidence suggests a role of SARS-CoV-2 in the pathogenesis of diverse thyroid disorders, such as subacute thyroiditis (SAT) or non-thyroidal illness syndrome (NTIS) which are common in SARS-CoV-2 infected patients [5]. Conversely, patients with a pre-existing thyroid dysfunction might be more susceptible to SARS-CoV-2 infection and more prone to complications [6]. Thus, particular attention must be reserved for COVID-19 patients with pre-existing thyroid diseases, but a routine thyroid assessment can be beneficial to all COVID-19 patients at admission, during hospitalization, and at follow-up checkups.

The mechanisms underlying this reciprocal relationship have not been completely elucidated, although a direct infection of the endocrine glands by the virus using angiotensin-converting enzyme 2 (ACE2) as a receptor for cellular entry as well as indirect proinflammatory (cytokine storm) immune-mediated damage could play a role [6]. Nonetheless, additional biomarkers and related pathways may explain, almost in part, the intermingled relationship between COVID-19 and thyroid dysfunction. In this context, selenium (Se), a trace microelement critical for the function of the thyroid gland, is emerging as a determinant for risk, severity, and clinical outcomes in SARS-CoV-2 infection [7,8]. In fact, Se is a potent antioxidant and plays a beneficial role in terms of the occurrence and severity of various viral infections (e.g., influenza, hepatitis), with the majority of its effects derived from its incorporation into selenocysteine (Sec), a component of selenoproteins [8].

In 1998, the World Health Organization estimated an adequate Se intake at 26 and 34 μg/day for adult females and males, respectively [9]. The recommended dietary allowance was subsequently revised to 55 μg/day by the Food and Nutrition Board at the Institute of Medicine of the National Academies [10], based on the amount of Se needed to maximize the action of the selenoprotein glutathione peroxidase (GPx), while Se deficiency was estimated to be equal to an intake < 40 µg/day [9,10]. On the other hand, the Tolerable Upper Intake Level for Se was set at 400 μg/day, given an increased risk for selenosis from an excessive Se dietary intake [7,10]. In 2014, the European Food Safety Authority in the European Union had established an adequate daily intake for Se at 70 µg [11]. Therefore, the dietary intake of Se remains difficult to estimate, as Se content and availability in soils varies widely across different geographical areas in the world, affecting its content in edible vegetables [7]. Conversely, Se levels in drinking water are generally low, so much so that this source is not considered a significant nutritional supply of this element [7]. Among foods, cereals, meat, fish, eggs, and dairy products are the major dietary sources of Se, where the intake depends on the amount of food in addition to the type consumed [7].

As far as SARS-CoV-2 infection is concerned, low soil Se levels and low daily Se intake have been associated with higher COVID-19 incidence, progression, and outcomes [12,13]. Moreover, COVID-19 patients had low blood Se levels associated with increased levels of oxidative stress and inflammatory biomarkers and lower circulating antioxidants [14,15]. Selenium supplementation (e.g., sodium selenite) may therefore represent an opportunity as it could serve as an adjunctive tool for the treatment of coronavirus infection for its anti-inflammatory, immunostimulatory, and antiviral actions, possibly preventing SARS-CoV-2-triggered thyroid aggravation in patients during and after COVID-19 [16,17].

Furthermore, innovative Se nanomedicine has shown that the production of Se nanoparticles (SeNPs), characterized by low toxicity and significant and selective effects obtainable with very small quantities (greatly reduced risk of toxicity in the face of high antiviral capability), may represent a promising opportunity [18,19]. In fact, SeNPs have been found able to reduce oxidative stress, promote apoptosis (killing cancer cells), modulate immune activity, and exert efficacious antimicrobial and antiviral activity, and as such, they may have potentially beneficial applications in the COVID-19 context [20,21,22].

This review aims to summarize the rapidly evolving available clinical data and mechanisms emerging in the relationship between SARS-CoV-2 and thyroid dysfunctions. In particular, the role of Se, critical for thyroid function, is discussed in view of the possible role of this element in the onset and development of the COVID-19 and as a mediator of the mutual interaction between SARS-CoV-2 infection and thyroid disease. References were identified by searching in Pubmed and Medline for original articles published in English from 1 March 2020 to 31 December 2021 by use of the MeSH terms “thyroid”, “thyroiditis”, thyroid disease”, “hypothyroidism”, “hyperthyroidism”, and “Graves’ disease”, in combination with the terms “COVID-19” and “coronavirus”. In addition, a search strategy was performed for the same period using the MeSH term “selenium” in combination with the terms “COVID-19” and “coronavirus” or the MeSH term “selenium” in combination with the terms “COVID-19”, “coronavirus”, thyroid”, “thyroiditis”, thyroid disease”, “hypothyroidism”, “hyperthyroidism”, and “Graves’ disease”. Articles and reviews cited in these references were also reviewed.

## 2. Bidirectional Interaction between Thyroid Dysfunction and COVID-19

Since the COVID-19 outbreak, increasing data resulting first from case reports and series followed by observational retrospective or cohort studies have emerged on the onset of thyroid dysfunction in patients during or after COVID-19 infection. Some valuable reviews have also attempted to improve knowledge on the relationship between COVID-19 and the thyroid gland.

So far, the guidelines on clinical management of the World Health Organization on COVID-19 infected patients do not recommend the evaluation of thyroid function. However, within this complex relationship, a range of biochemical, immunological, and epidemiological evidence could support the role of SARS-CoV-2 in leading to short-term and reversible thyroid dysfunction [23] (Table 1). Conversely, a number of elements still need to be clarified within this association mainly because of the lack of completeness of the data, the difficulty of comparing studies characterized by differences in study design, sample size, populations studied, number and type of thyroid hormone measurement, confounding factors considered, and the possibility of publication bias (Table 2).

**Figure 1:**
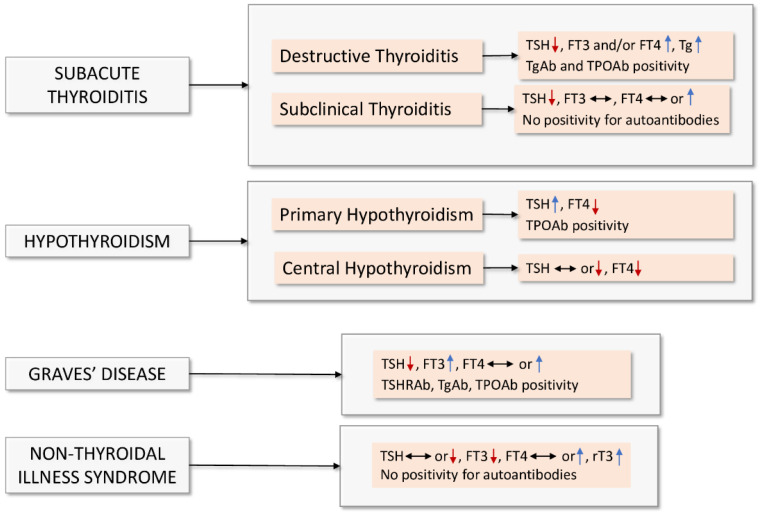
Main features of thyroid diseases potentially involved in SARS-CoV-2 infection. The arrows in the figure represent an increase or decrease in the serum concentration of thyroid parameteres. Abbreviations: FT3—free triiodothyronine; FT4—thyroxine; rT3—reverse triiodothyronine; Tg—thyroglobulin; TgAb—thyroid antithyroglobulin antibody; TPOAb—thyroid peroxidase antibody; TSH—thyroid-stimulating hormone; TSHRAb—autoantibodies against thyroid-stimulating hormone receptor.

As evidenced by Scappaticcio and coauthors [73], the published studies have reported that COVID-19-related disorders may mainly manifest as SAT, NTIS, and hypothyroidism, which are characterized by specific thyroid hormone parameters depicted in Figure 1. SAT, also called de Quervain thyroiditis, is a self-limited, inflammatory disorder characterized by neck pain and systemic symptoms [74], with a picture including suppressed thyroid-stimulating hormone (TSH), high free thyroxine (FT4) and thyroglobulin, and absence of TSH receptor antibodies [41,64] (Figure 1). Thyroid dysfunction presenting as typical painful SAT was principally diagnosed in female patients with mild to moderate COVID-19 disease, as described in several case reports [30,31,32,33,34,35,36,37,38,39] and reported in up to 20% of noncritically ill hospitalized patients [41], at a much higher incidence than that recorded in the general population [75]. SAT is presumed to be caused by a viral infection or a postviral inflammatory process in genetically predisposed individuals [24,40]. For certain viruses, direct evidence based on epidemiological and serological data of viral infection on the thyroid is available, although it is not clear if they are responsible for thyroid diseases (reviewed in [24]) (Table 1). The onset of SAT may occur concurrently with COVID-19 diagnosis [30,35,39] or, in most cases, after a few weeks as a late complication of SARS-CoV-2 infection [31,32,33,34,36,37,38]. The clinical course of SAT generally comprises multiple phases: first is the thyrotoxicosis (lasting a period of weeks or months) in the majority of patients, followed, in some cases, by hypothyroidism, while treatment with anti-inflammatory agents or corticosteroids in the most severe cases, generally resulted in rapid clinical resolution [31,35,36,37,38,39,40,76,77]. Although it is conceivable that SARS-CoV-2 could be associated with SAT, in none of the patients, the presence of the virus was demonstrated in the thyroid tissue. Additionally, the retrospective or single-center design of most of the studies performed precluded clarifying whether chronic thyroiditis can develop after COVID-19 resolution (Table 2).

In patients with COVID-19 requiring high intensity of care, atypical painful thyroiditis was reported, which was negative for thyroid autoantibodies and characterized by lymphopenia in an underlying setting of NTIS [47]. On the other hand, in the study of Lui et al. [48], none of the patients, most of whom had the mild disease at admission, presented with overt symptoms of thyroiditis; however, in three of them, positivity for autoantibodies was suggestive of thyroid autoimmunity (Figure 1).

Thyrotoxicosis appears to be clinically relevant, as revealed by the high prevalence of atrial fibrillation and the degree of TSH decrease, an increased mortality risk, and the length of hospitalization in COVID-19 patients [41,47] (Table 1). Of note, 16% of patients with overt thyrotoxicosis developed thromboembolic events at twice the rate of COVID-19 subjects admitted to nonintensive care units (ICU) [41]. In contrast, no association was found between suppressed TSH concentration and mortality in patients hospitalized for moderate-to-severe COVID-19 disease [48]. Reduced levels of TSH were also shown in patients with nonsevere COVID-19 compared with healthy individuals, although the cross-sectional design did not allow to infer a causal relationship [49] (Table 2).

NTIS, which generally presents with a decrease in triiodothyronine (T3) concentration in a context of normal thyroid function (Figure 1), was reported in around 15–34% of ward patients [49,50,51,52,64] and up to 60% in patients admitted to the ICU [49]. A pattern of thyroid hormone alterations compatible with NTIS can be associated with severe pneumonia and multiple COVID-19 adverse events such as clinical deterioration, supplementary oxygen, and prolonged hospitalization, in accordance with the association of this condition with critical illness and treatment in ICU [48,50,51,78] (Table 1). Furthermore, reduced free triiodothyronine (FT3) concentration could play a crucial role in predicting all-cause mortality in patients with severe COVID-19 [51], although NTIS may also manifest in individuals with mild to moderate COVID-19 not requiring ICU [48] and is transient once the infection is cured [50,64]. Importantly, the decrease in T3 concentration observed in the acute phase of critically ill patients and attributable to reduced 5’-deiodinase activity and increased concentration of reverse T3 by 5-deiodinase enzyme would initially lead to an increase in TSH and thyroxine (T4), followed by central suppression of the thyroid axis and consequent reduction in T4/FT4 and TSH levels in the most severe chronic conditions, indicating that this disorder could represent an adaptive response to preserve energy during systemic illnesses [49,79,80].

Despite the restricted number of cases, according to a meta-analysis based on eight observational studies, thyroid disease appears to be associated with an enhanced risk of severe COVID-19 infection [53]. Indeed, the cross talk between thyroid hormone action and the immune system has been established in physiological and pathological settings, with thyroid hormones being modulators of immune responses [54,55] (Table 1). An overactive innate immune response has been recognized to contribute to pathogenesis in COVID-19 patients requiring hospitalization, though the lack of studies on nonhospitalized patients does not make it possible to define the role of the innate immune system during SARS-CoV-2 infection [81]. Vassiliadi et al. [49] included appropriate control groups showing that the prevalence of the thyrotoxicosis pattern was not significantly different between critically and noncritically ill patients with or without SARS-CoV-2. Regarding NTIS, the most important factor is probably the severity of illness since significant differences in NTIS patterns were encountered between patients admitted to the ICU and patients in the ward, with similar frequencies between SARS-CoV-2 in positive and negative cases [49]. Therefore, as highlighted in a recent systematic review, which showed that most studies evaluated thyroid function in critically ill patients, the observed thyroid profile, especially NTIS, could be not specifically related to COVID-19 [69] (Table 2).

During the past SARS epidemic, a substantial number of patients displayed abnormalities in thyroid function, as described by histopathological findings in autoptic studies [25,26,28,29]. Additionally, in the study of Leow et al. [27] performed on SARS-CoV survivors three months following recovery, 6.7% of subjects had developed hypothyroidism biochemically, primarily of central etiology, which spontaneously remitted, whereas the patient with primary hypothyroidism required permanent L-thyroxine replacement (Table 1, Figure 1). Conversely, neither the abnormalities in the thyroid follicular morphology, except for chronic inflammation [82] nor the significantly reduced levels of T4 were found in patients who died of SARS-CoV-2 [83]. Furthermore, primary hypothyroidism has only been reported in a few hospitalized patients with COVID-19 [41] and, in some cases, was due to concomitant autoimmune thyroiditis [47,48,66,70] (Table 2). This finding suggests that SARS-CoV-2 could act as a trigger of latent or new-onset autoimmunity, in accordance with the data indicating a multifactorial etiology, including susceptibility genes, iodine, medications, smoking, and infections in autoimmune thyroid diseases [84].

Multiple mechanisms have been proposed as contributors to pathogenic pathways resulting in thyroid follicular cell destruction [85]. Thyroid damage could result from a direct effect of SARS-CoV-2 [23]. As widely ascertained, SARS-CoV-2 is internalized into host cells mainly through ACE2 and transmembrane protease serine 2. Importantly, ACE2 expression has been reported in most tissues at the highest levels in thyroid follicular cells, and this could provide a plausible mechanism for the pathophysiology of thyroid dysfunction following the viral infection [23,56]. An alternative pathophysiological pathway involves the binding of thyroid hormones to the integrin receptors, which have been hypothesized to facilitate SARS-CoV-2 internalization and result in the regulation of the transcription of genes involved in antiapoptotic and angiogenic properties and the enhancement of cell proliferation [58,59] (Table 1).

An indirect mechanism has also been speculated. In COVID-19 patients, in the event of dysfunctional and exaggerated immunological response, several proinflammatory cytokines, including interleukin-6 (IL-6) that covers a crucial role in acute inflammation, may give rise to a cytokine storm, which causes a severe lung and even immunopathological systemic damage [41,80]. This hyperinflammatory status might also lead to thyroid dysfunction [65,80], consistently, at least partly, with the immune activation occurring in the pathogenesis of thyroid autoimmunity [60,61] and NTIS [62,63]. Indeed, thyrotoxicosis seen in affected subjects appears to be closely associated with higher levels of IL-6, IL-8, IL-10, IL-15, granulocyte macrophage colony-stimulating factor, and interferon-gamma (IFN-γ)-induced protein 10, supporting the theory that thyroid gland inflammation could be triggered and sustained by the flood of cytokines and chemokines in the serum [41,64,65]. Likewise, Gao et al. [51] found that, in a pattern of NTIS, FT3 was significantly and inversely associated with C-reactive protein (CRP), a general marker of inflammation, and IL-6 in nonseverely ill patients and survivors, but not in nonsurvivors, indicating that the inflammatory response may involve different manifestations of NTIS based on the different COVID-19 stage. The transient finding of low TSH with normal FT4 and low FT3 levels, along with the inverse correlation of TSH and FT3 with CRP, IL-6, and cortisol, further suggests that the cytokine storm produces a combined effect both centrally, with a decrease in TSH, and at the peripheral level, with the reduced conversion of FT4 to FT3 [41]. Instead, within SAT, when TSH suppression occurs without changes in thyroid hormone levels, hyperinflammation affects the hypothalamic-pituitary axis exclusively at a central level [65]. Although these results overall hint that thyroid dysfunction in COVID-19 patients is likely related to severe inflammation [41], it cannot be excluded that the observed decrease in TSH concentration could be the consequence of concomitant treatment with glucocorticoids, which may affect the rate of TSH secretion [50,51]. Similarly, heparin, which is largely used in COVID-19 patients, may interfere with free thyroid hormone assays; in fact, heparin leads to increased lipoprotein lipase activity generating nonesterified fatty acids that in turn displace T3 and T4 from binding proteins [69,86] (Table 2).

Notably, IL-6 could also induce the onset or recurrence of hyperthyroidism in patients with Graves’ disease (GD), probably stimulating TSH receptor expression as well as promoting inflammation in GD [87,88]. GD relapse has been documented in patients after the clinical onset of COVID-19, indicating that GD could have been reactivated by the viral infection via an immunoinflammatory process [66,80]. On the other hand, a large retrospective cohort study aimed at evaluating the impact of pre-existing hypothyroidism on COVID-19 outcomes revealed that hypothyroidism was not associated with increased risk of morbidity and mortality, and the authors did not recommend any additional precautions [71].

## 3. Role of Selenium in COVID-19: Clinical Significance and Implications for Therapy

**Figure 2:**
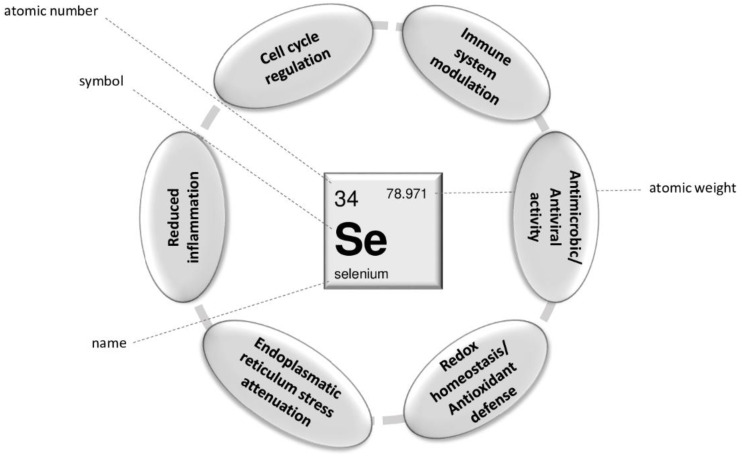
Major pathways modulated by selenium.

Selenium is an essential trace element in humans, exerting multiple beneficial actions at a cellular level that are principally due to the biological role of selenoproteins [89]. Selenoproteins (in humans, identified 25 proteins belonging to this family) are fundamental both for the developmental processes and for the pathological evolution of cells [90]. Among these, only two are extracellular, the selenoprotein P (SELENOP), which contains most of Se in the plasma (10 Sec residues), and the extracellular glutathione peroxidase 3 (GPx3) [90]. Overall, families of selenoproteins include GPxs, iodothyronine deiodinases (DIOs), thioredoxin reductases (TrxRs), and methionine sulfoxide reductase B1. These redox enzymes exploit the chemical properties of Se for antioxidant protection, redox homeostasis, and endoplasmic reticulum stress [13]. GPx1-4 and TrxR are involved in the protection against oxidative stress and oxidant-driven cell death [89]. Additionally, GPx and TrxR enzymes also play a central role in nitrosative stress responses, metabolizing nitrosothiols and peroxynitrite, two major reactive nitrogen species [91]. GPx3 is considered a tumor suppressor, GPx2 seems to act at the very early stages of cancer, while selenoprotein H is a key regulator for the cell cycle, indicating the role of selenoproteins in cancer could be both preventive and therapeutic (Figure 2) [89]. Selenium and selenium-containing compounds attenuate inflammatory signaling pathways, with the four isoforms of GPx reducing the high levels of free radicals produced during inflammation and cytosolic GPx4 inhibiting interleukin-driven-nuclear factor (NF)-kB activation and leukotriene biosynthesis [89,92].

Selenoprotein levels react differently to blood Se decrease, in accordance with the so-called “hierarchy of selenoproteins”. GPx1 is considered at the lowest level in the hierarchy since it rapidly decreases in case of Se restriction and its resynthesis occurs very late after Se repletion [93]. Other selenoproteins, such as GPx2, GPx4, DIOs, and TrxRs, rank high in the hierarchy because of their high mRNA stability during Se restriction and rapid biosynthesis at Se recovery [93]. Notably, it is crucial to maintain an adequate level of Se, being decisive in the redox homeostasis; thus, both deficiency and excess can be dangerous for human health [94]. This implies that the range of adequate serum Se is narrow (between 90–120 µg/L, sufficient to achieve maximum GPx3 activity and blood selenoprotein P concentration), while below and above this range, the risk of adverse effects increases [7,94].

In recent times, the possible link between COVID-19, Se intake, and the potential mechanisms involved have been investigated [13]. In fact, since therapeutic approaches against COVID-19 infection are still scarce, the importance of nutritional strategies aimed at maintaining a healthy immune system has been particularly highlighted. In this context, Se is one of the elements to be taken into careful consideration because of the incorporation of Sec in the polypeptide chain of selenoproteins. The hypothesis proposed on the role of Se in SARS-CoV-2 infection is mainly based on the increase in oxidative stress, a severe reduction in blood Se levels, and decreased concentration of SELENOP and GPx3 in COVID-19 patients [95]. Of note, GPx1, and possibly also TrxR1 and selenoprotein F, are substrates of M^pro^, the main cysteine protease of SARS-CoV-2, and the proteolytic cleavage of these antioxidant molecules results in increased oxidative stress, NF-κB activation, and proapoptotic signaling [96].

Selenium deficiency has been recognized as a risk factor for viral infections [97]. The immune system relies on several selenoproteins such as Gpx4, which is fundamental in T cell immunity by preventing ferroptotic cell death, selenoprotein K, which is involved in proliferation, migration, cytokine secretion of immune cells and protection against pathogens, and the selenoprotein S, which mitigates endoplasmic reticulum stress during macrophage activation [98]. Even though Se deficiency is a rare condition, suboptimal levels are rather frequent, and an association between low Se levels and predisposition to viral infection has been speculated. Specifically, Se deficiency appears to increase oxidative stress and mutation rate in the viral genome, allowing a greater susceptibility to severe viral infections [97] (Figure 2).

Selenium levels were found to be inversely associated with the incidence of various viral infections around the world. A set of interesting studies published by Beck’s laboratory in the 1990s showed that host Se deficiency increased the virulence of RNA viruses such as coxsackievirus B3 and influenza A [97,99]. Additionally, infection in a Se-deficient animal unable to produce sufficient antioxidant selenoproteins for its own protection resulted in the virus mutating to a virulent form that caused more severe pathology [97,99]. Accordingly, Se deficiency functions as an independent predictor of survival in patients with HIV-1 infection [100]. Of particular importance is the inverse relationship between blood Se levels and the cardiomyopathy called Keshan disease (caused by coxsackievirus B3) endemically affecting northeast China regions [99]. Keshan disease showed seasonal variability, later found to be accompanied by the viral infection of Coxsackie B3 enterovirus. When the population was supplemented with Se, the incidence of Keshan disease decreased dramatically [99,101].

On the other hand, the evidence provided by respiratory viruses has shown that changes in redox homeostasis, endoplasmic reticulum stress, and inflammation pathways may be modulated by Se or selenoproteins, and this could also occur in infection from SARS-CoV-2 [13]. Coronaviruses, the family to which SARS-Cov-2 belongs, have crucial influences on a large number of RNA-related biological pathways during cellular infection [102]. In particular, the influence of SARS-CoV-2 on the non-sense-mediated RNA decay, a mechanism that functions as a cell quality-control mechanism ensuring the translational accuracy of mRNA transcripts and is believed to be involved in the regulation of selenoprotein mRNA levels, has been described [103,104]. Hence, SARS-CoV-2, interfering with the metabolism of selenoproteins and consequently with their action, could lead to adverse effects, especially in subjects characterized by low circulating levels of Se.

Taking into account the history of viral infections related to Se deficiency, the relationship between Se deficiency and the SARS-CoV-2 infection has been recently explored.

In a systematic review including a total of 11 studies, nine of them suggested lower blood Se levels associated with worse outcomes, although the other two studies found no significant association between Se and COVID-19 [105]. In one of the included studies, urinary Se level was higher in severe and fatal cases compared with nonsevere and recovered patients. Overall, Se deficiency was thus generally associated with worse outcomes, and COVID-19 patients exhibited Se levels lower than healthy subjects [105].

It is interesting to note that in China, there are deficiencies of Se in the belt extending from the northeast to the southwest of the country, although there are also areas with an exceptionally high Se content in the environment, so groups of people living in the same country have very low or very high Se levels [106]. Analyzing data from Chinese provinces and municipalities with more than 200 cases of COVID-19 and cities with more than 40 cases, in areas with high levels of Se in the diet, patients recovered better from the virus [107]. For instance, in the city of Enshi in Hubei Province, which is characterized by the highest dietary Se intake in China, the cure rate (percentage of COVID-19 patients considered “cured”) was almost three times higher than the average for all other cities in Hubei Province. In contrast, in Heilongjiang Province, where Se consumption is among the lowest in the world, the death rate from COVID-19 was nearly five times higher than the average of the other provinces [107]. In support of their thesis, the authors found that the COVID-19 cure rate was significantly related to Se levels, as measured by the amount of the element measured from patients’ hairs in 17 cities outside Hubei [107].

The correlations identified were convincing, especially based on previous research on Se and infectious diseases. As such, a careful and in-depth evaluation of the role of Se in COVID-19 is certainly warranted and can assist in making day-to-day public health decisions. A significant relationship between Se levels and COVID-19 cure rate has been documented; however, it is also important not to overestimate this finding since the researchers were unable to work with the data at the individual level and adjust the association for possible confounders such as the age and other underlying diseases of the patients [107].

As previously reported, Se is an efficient NF-kB inhibitor; it thus exerts immune-regulatory and anti-inflammatory actions and could rebalance the host response [108,109]. NF-kB is generally considered antiapoptotic, although it may also promote apoptosis in response to cellular stress [110]. In particular, RNA viruses such as influenza virus are able to activate NF-kB and subsequent host innate immune responses against viral infection and, at the same time, incorporate the NF-κB pathway into their own life cycles and pathogenesis [111,112]. In SARS-CoV infection, NF-kB is strongly responsible for the pulmonary lesions observed [113], and it promotes the cytokine storm associated with severe COVID-19 [114].

So far, no clinical trials have been designed to evaluate the administration of Se to COVID-19 patients; however, a series of animal and human studies suggest that optimal Se levels or Se administration can prevent excessive cytokine production [115,116,117]. Furthermore, Se supplementation in mechanically ventilated patients appears to improve illness severity and reduce the incidence of ventilator-associated pneumonia [118,119]. An adequate Se intake is essential to improve resistance to viral infection, and its administration at an early stage can be decisive for restoring the host antioxidant efficacy and reducing SARS-CoV-2 cellular damage and alteration of metabolic processes [120].

Sodium selenite supplementation has been hypothesized to prevent COVID-19 infection due to its ability to oxidize thiol groups in the virus protein disulfide isomerase, thereby inhibiting virus entrance into healthy cells [121]. Sodium selenite is more bioavailable than Se and can cross the blood–brain barrier, rapidly restoring cellular DNA synthesis and antioxidant potential, according to the mechanism already suggested for other viral infections [109]. During the COVID-19 pandemic, several studies reported lower blood Se levels in infected patients than in control subjects from different ethnic groups and a positive correlation between Se deficiency and reduced survival [14,95,122,123,124]. In particular, Zhang et al. reported a significant association between reported cure rates for COVID-19 and Se status, opening up to Se supplementation as a tool against clinical manifestations and complications of COVID-19 [107].

Therefore, Se-enriched food and supplementation (e.g., sodium selenite) could be considered in populations with suboptimal Se in their diet/environment to prevent SARS-Cov-2 infection, as well as measurement and monitoring of Se in hospitalized and symptomatic outpatients, taking care to avoid adverse effects related to excessive Se (supplementation may potentially increase the risk of certain diseases and toxicity in individuals with a pre-existing adequate or high status) or in other specific conditions (e.g., patients with type 2 diabetes) [16,109,121,125,126]. For these reasons, a combined assessment of Se, SELENOP, and zinc (Zn) could serve as a reliable biomarker of survival in COVID-19 patients, contributing to a better stratification of patients affected by COVID-19 (or other similar infectious diseases) and suggesting that a personalized adjuvant supplementation of microelements may help clinical care [123,127].

Ebselen (a Se-based organic complex and a GPx mimic, with anti-inflammatory, antioxidant, and cytoprotective properties and antibacterial and antiviral effects) shows inhibitory activity against M^pro^, interrupting virus replication and transcription, and as such, it is a possible novel therapeutic option to face COVID-19 [128,129]. Also, Ebselen appears to efficaciously inhibit PL^pro^ (another enzyme critical for the virus replication by processing the viral polyprotein into mature and functional subunits, and by which the SARS-CoV-2 virus antagonizes the host’s antiviral innate immune response) through a covalent binding with the sulfhydryl group of the Cys112 residue in the catalytic triad [130].

In this scenario, the innovative Se nanomedicine has also shown that the production of SeNPs could be employed in a number of applications. NPs are characterized by low toxicity, and significant and selective effects are obtainable with very small quantities, namely a substantially reduced risk of toxicity in the face of a high antiviral capacity [18,19]. Recent in vitro data have reported that treatment with the antiviral drug oseltamivir, in combination with SeNPs, can increase cell viability to prevent Enterovirus 71 infection and reduce apoptosis (via the Bax signaling pathway) [131]. Additionally, the combined administration of SeNPs with a conventional hepatitis B vaccine induces a better immune response (activation of lymphocyte proliferation, increased IFN-γ expression, and induction of the Th1 response), supporting the ability of SeNPs to maximize the immune response and increase the efficacy of the vaccine [132]. In general, SeNPs have been found able to reduce oxidative stress, promote apoptosis (by killing cancer cells), modulate immune activity, and exert effective antimicrobial and antiviral activity and, consequently, have potentially beneficial effects also for their application in the COVID-19 clinical setting [20,21,22]. Another intriguing development in this field is the nano biofortification strategy by which edible plants can be enriched with essential nutrients (e.g., Se through fertilization) for human health and for addressing diseases such as COVID-19 [133]. Even as an indirect tool against COVID-19, SeNPs deposited on polyester fabric via a flat screen-printing technique showed virucidal properties against the SARS-Cov-2 virus, inhibiting its growth, with an efficiency of 87.5%, finding a potential use for protective clothing applications [134].

### The Relationship between Selenium Status and COVID-19 Vaccination

Selenium supplementation has been demonstrated to enhance both cellular and humoral immune responses, which are both crucial against bacterial and viral infections [135]. In particular, high Se intake is involved in adaptive immunity by activating the proliferation and differentiation of cluster of differentiation (CD)4+ T cells into T-helper-1 (Th1), leading to increased expression of IFN-γ and CD40 ligand [136,137]. Selenium levels also influence the innate immune response through the activation of macrophages by promoting their transition from the classical (M1) phenotype to the alternative (M2) pathway, which is implicated in the resolution of inflammation [138]. In addition, selenoproteins are essential for the normal function of T cells, which are particularly sensitive to oxidative stress [139]. Indeed, GPx4 is a key scavenger of phospholipid hydroperoxides, and the genetic deficiency of GPx4, although it does not alter the normal thymic development of T cells, causes lipid peroxidation and subsequent death by ferroptosis of CD4+ and CD8+ T cells. Conversely, the addition of Ebselen restores the dose-dependent survival of Gpx4-deficient T cells [140]. Similarly, Yao et al. (2021) recently found that supplementation of Se resulted in increased GPx4 synthesis in T follicular helper (T_FH_) cells, a subset of CD4+ T cells particularly susceptible to ferroptosis, thereby potentiating T_FH_ function in both immunized mice and young adults who received influenza vaccination [141]. On the other hand, in a previous randomized, double-blinded, placebo-controlled clinical trial, the effects of Se supplementation on both cellular and humoral immune responses to flu vaccine were tested in healthy older subjects aged 50-64 years [142]. The authors observed both beneficial (dose-dependent increase in T cell proliferation, IL-8, IL-10, and IFN-γ) and detrimental (lower granzyme B content of CD8 cells and suppressed synthesis of tumor necrosis factor-alpha, TNF-α) effects of Se supplementation on cellular immunity to influenza, supporting the hypothesis that concentration of Se has a narrow effective range [142]. Interestingly, the incorporation of SeNPs in diet and vaccine significantly increased the expression of IL-2, IL-6, and IFN-γ in blood cells in a dose-dependent manner, enhancing vaccine efficacy against highly pathogenic avian influenza virus H5N1A in chicken [143]. In a recent observational study aimed at investigating the potential correlation between Se status and antibody response following COVID-19 mRNA (BNT162b2) vaccination, 126 health care workers were administered two consecutive doses of vaccines and followed for up to 24 weeks [144]. The humoral immune response measured as SARS-CoV-2 IgG titers and neutralization potency of the serum on SARS-CoV-2 spike protein at four time points (0, 3, 6, and 24 weeks) was compared between participants with self-reported Se supplementation and those not reporting recent Se supplemental intake, with no difference detected. Furthermore, the correlation of SARS-CoV-2 titers with baseline total Se, the concentration of SELENOP, and GPx3 activity (all categorized per tertiles) was evaluated in both groups, and no significant differences were observed, except for a slight U-shaped difference for SELENOP at the second time point [144]. Nonetheless, all together, these findings suggest a role of Se on the immune system, opening interesting perspectives for further studies exploring the effects of baseline Se status on the immune response following COVID-19 vaccination with possible consequences on thyroid pathophysiology and whether Se supplementation before, concurrently, or after the vaccine, in subjects with an inadequate Se status may benefit the immune system and protect against thyroid dysfunction.

## 4. Conclusions

The accumulated evidence suggests an influence of SARS-CoV-2 infection on the thyroid gland, although multiple thyroid pictures have been described. SAT appears as the most common thyroid-related clinical syndrome associated with COVID-19, and since it may occur regardless of the severity of COVID-19 as an underestimated manifestation of infection, its early diagnosis and management would be suggested. Some authors also recommend clinical and biochemical monitoring after discharge since SAT may delay the recovery in these patients. Careful monitoring of thyroid function is especially recommended in individuals with previous autoimmune thyroid diseases. Meanwhile, the possible occurrence of thyroid dysfunction should always be considered a possible complication during and after COVID-19 infection, with serum FT3 levels providing a valuable tool to stratify the management of severe COVID-19. The inconsistent clinical manifestations of thyroid abnormalities in patients with COVID-19, as well as the great variation in the reported incidence of thyroid dysfunction, primarily depends on the definition of thyroid disease, the type of population studied, the hormonal tests performed, the timing of assessment, and the concurrent medications. The exact mechanisms underlying thyroid dysfunction during or after SARS-CoV-2 infection are not completely elucidated and warrant further research, while multicentre longitudinal studies, including large cohorts and complete hormonal test assessments, are needed to verify the long-term consequence of COVID-19 on hypothalamic-pituitary-thyroid axis. Additionally, although there is currently no strong evidence on the role of pre-existing thyroid disease in increasing the risk of SARS-CoV-2 infection, this aspect should be further investigated considering the rapid, widespread diffusion and the new variants of the virus.

The thyroid is among the human tissues with the highest Se content. Selenoproteins that exert their actions in cellular antioxidant systems and redox modulation, such as GPxs and TrxRs, are involved in the protection of the thyroid gland from an excess of hydrogen peroxide and reactive oxygen species generated by the follicles during the production of thyroid hormones [145] (Table 3). The three iodothyronine deiodinases, the enzymes controlling thyroid hormone metabolism through hormone activation/inactivation, are selenoproteins (Table 3). Selenium can also modulate the immune response in patients with autoimmune thyroiditis and retains insulin-mimetic properties, an effect likely determined by the stimulation of tyrosine kinases involved in the insulin signaling pathway. Therefore, Se deficiency has been associated with thyroid disorders (e.g., subclinical hypothyroidism, autoimmune thyroiditis, and Hashimoto’s thyroiditis), and beneficial effects of Se supplementation have been reported, although recommendations for Se supplementation currently only concern patients with Graves ophthalmopathy [7].

An unpublished clinical trial using Se supplementation (NCT04751669; adjuvant treatment of Selenious Acid infusion at 2000 µg on day 1, followed by a continuous infusion of Selenious Acid at 1000 µg daily on days 2–14 for moderately ill, severely ill, and critically ill COVID-19 patients to decrease overall mortality), and two unpublished studies on Se plus zinc supplementation (1-NCT04323228: 15 μg/day Se and 7.5 mg/day Zn for 14 days to assess effects on ferritin and cytokine storm biomarkers—CRP, IL-6, TNF-α, monocyte chemoattractant protein 1, total leukocyte count, differential count, and neutrophil to lymphocyte ratio; NCT04751669: 10 mg/day Zn and 110 μg/day Se for 14 days in COVID-19 outpatients to evaluate the possible reduction in 3 month-hospital admissions) are currently ongoing [150].

To the best of our knowledge, no results are available yet, nor have any studies been designed to assess the role of Se in thyroid complications in COVID-19 patients. Nevertheless, given the mutual relationship between thyroid and COVID-19 (mainly attributable to pathways related to oxidative stress/redox balance and inflammation) and Se being critical both for thyroid function and for the susceptibility and prognosis of SARS-CoV2 infection, ongoing and future trials are expected to discover whether restoration of an adequate Se status may be useful in preventing and addressing COVID-19, avoiding adverse effects. Selenium can be a very dangerous element, and everything depends on the chemical form of selenium and its concentration. Therefore, it is very important to gradually study its effect on living organisms [7]. Moreover, future investigations could establish whether appropriate Se levels will help avoid thyroid complications that can contribute to worsening outcomes in these patients.

## Figures and Tables

**Table 1:** Summary of evidence supporting the association between COVID-19 and thyroid dysfunction.

Clues	Levels of Data	Main Features/Findings	Publication Type	Reference
Evidence for the presence of certain viruses or their components in SAT and autoimmune thyroid diseases	Epidemiological; serological, direct evidence	Retroviruses (HFV) and mumps: direct evidence in SAT. Retroviruses (HTLV-1, HFV, HIV, and SV40) direct evidence of GD. HTLV-1, enterovirus, rubella, mumps virus, HSV, EBV, and parvovirus: direct evidence in Hashimoto’s thyroiditis	Review	[24]
SARS-CoV detected in many endocrine organs, including the pituitary gland	Histological; serological	China; four patients died from SARS (three males aged 25, 38, and 57 years, and a 62-year-old female), four control patients (two males, 32 and 52 years old, and two females, 28 and 68 years) died of cardio-/cerebrovascular disease or ectopic pregnancy.	Case-control (postmortem) study	[25]
	Histological; cellular; molecular	China: 18 patients died from suspected SARS, 22 died from confirmed SARS, 65 T lymphocyte counts in 65 confirmed and 35 misdiagnosed SARS cases	Retrospective (postmortem) study	[26]
Possibility of transient subclinical thyrotoxicosis or reversible hypothyroidism within a hypothalamic-pituitary-adrenal axis dysfunction occurring associated with SARS-CoV	Molecular	Singapore; 61 survivors of SARS (aged 21 years and above, 39.9% with hypocortisolism after a 1-year follow-up)	Prospective cohort study	[27]
Significant damage to the follicular and parafollicular cells of the thyroid detected in SARS-CoV nonsurvivors	Histological	China; five thyroid samples from patients with SARS (four males, one female, 24–50 years old); 10 thyroid samples from controls of comparable age	Case-control study (postmortem) study	[28]
Significant reduction in the number of positive cells and the staining intensity of immunoreactivity for TSH in the adenohypophysis of patients with SARS-CoV compared with controls	Histological	China; five pituitary samples from patients with SARS (four males, one female, 24–51 years old); five pituitary samples from controls of comparable age	Case-control (postmortem) study	[29]
Both direct viral and postviral manifestations of COVID-19 associated with SARS-CoV related thyroiditis	Clinical; imaging; molecular	41-year-old Caucasian woman (mild COVID-19)	Case study	[30]
	Clinical; imaging; molecular	Italy; an 18-year-old woman (mild COVID-19).	Case study	[31]
	Clinical; imaging; molecular	Italy; four females (29–46 years), one patient hospitalized due to atrial fibrillation	Case series study	[32]
	Clinical; imaging; molecular	Mexico; a 37-year-old woman (mild COVID-19)	Case study	[33]
	Clinical; imaging; molecular	India; a 58-year-old man (mild COVID-19)	Case study	[34]
	Clinical; imaging; molecular	Italy; a 37-year-old woman hospitalized at the COVID-19 department	Case study	[35]
	Clinical; imaging; molecular	Italy; a 43-year-old woman (mild COVID-19)	Case study	[36]
	Clinical; imaging; molecular	Norway; a 45-year-old woman (mild COVID-19); a 45-year-old woman (mild COVID-19)	Case reports	[37]
	Clinical; imaging; serological; molecular	United States; a 41-year-old woman (mild COVID-19)	Case study	[38]
	Clinical; imaging; serological; molecular	Iran; six patients (four women, two men), 26–52 years old (IgG and IgM positive for COVID-19, history of family members’ hospitalization due to COVID-19 pneumonia in 3 out of 6 cases)	Case series	[39]
	Clinical; imaging; instrumental; molecular	Myanmar; a 34-year-old man (mild COVID-19)	Case study	[40]
The prevalence of SAT in COVID-19 patients is higher than that in the general population	Epidemiological; molecular	Italy; 287 noncritically ill patients (193 men, median age 66 years)	Retrospective single-center study	[41]
NTIS is associated with critical illness and poor outcomes in patients with pneumonia, acute myocardial infarction, chronic renal failure, cirrhosis	Epidemiological; clinical; molecular	China; 503 hospitalized patients (mean age 63 years) with community-acquired pneumonia	Retrospective single-center study	[42]
	Epidemiological; instrumental; molecular	China: 2459 patients with AMI diagnosis (529 patients with low T3 syndrome, 529 euthyroid patients, >18 years)	Prospective cohort study	[43]
	Epidemiological, molecular	NTIS is ubiquitous in critical illness, while T3 replacement in this condition remains controversial.	Review	[44]
	Epidemiological; molecular	17 studies (14 cohort, three cross-sectional); 4593 patients with CRF, mean age 62 years	Systematic review and meta-analysis	[45]
	Epidemiological; molecular	China; 385 patients: with cirrhotic portal hypertension, mean age 56.5 years.	Prospective cohort study	[46]
The inverse relationship between TSH and FT3 levels and clinical severity in COVID-19 patients	Epidemiological; clinical; molecular	Italy; 287 noncritically ill patients (193 men, median age 66 years	Retrospective single-center study	[41]
	Epidemiological; clinical; molecular Clinical; imaging; molecular	Italy; 93 COVID-19 consecutive patients admitted to HICUs in 2020, 101 consecutive patients admitted to HICUs in 2019, and 52 COVID-19 patients admitted to LICU in 2020.	Prospective (COVID-19 patients); retrospective (controls)	[47]
	Epidemiological; clinical; molecular	Hong-Kong; 191 consecutive COVID-19 patients (84.3% mild, 12.6% moderate, 3.1% severe).	Prospective cohort study	[48]
	Epidemiological; clinical; molecular	Greece; 102 consecutive COVID-19 patients (41 admitted in the ICU, 46 admitted in the ward, 15 outpatients)	Prospective cohort study	[49]
	Epidemiological, molecular	China: 50 COVID-19 patients, 54 healthy patients/50 patients with pneumonia	Case-control study	[50]
	Epidemiological, clinical, molecular	China: 100 patients (66 critically ill)	Retrospective single-center study	[51]
	Epidemiological, clinical, molecular	United Kingdom; 456 patients (334 (73.2%) diagnosed with COVID-19, mean age 66.1 years)	Prospective cohort study	[52]
Thyroid disease are associated with COVID-19 severity	Epidemiological, molecular	6 studies (8 retrospective cohort, 2 case series); 2169 COVID-19 patients	Systematic review and meta-analysis	[53]
Thyroid hormones are involved in different aspects of innate and adaptive immune responses	Cellular	Genomic and nongenomic mechanisms by which T3 and T4 modulate the activity of macrophages and leukocytes; (innate immune response); natural kill cells (adaptative and innate immune response), and lymphocytes (adaptive immune response)	Review	[54]
	Cellular	Cellular and molecular signaling pathways are involved in the cross talk between THs and innate immune functions (neutrophils, natural killer cells, monocytes–macrophages, and dendritic cells)	Review	[55]
ACE2 and TMPRSS2 mRNA are highly expressed in the thyroid, suggesting a possible direct action of SARS-CoV-2 on the gland	Tissue; cellular	15 thyroid samples were obtained from the disease-free tissue of patients who underwent thyroidectomy for a nodular goiter (12 women and three men); two primary cultures of normal thyrocytes	In vitro ex vivo study	[56]
	Tissue	ACE2 and TMPRSS2 expression levels derived from the human protein atlas and genotype tissue expression	Review	[57]
Binding of thyroid hormones to the membrane integrin receptor which could be implicated in the transmission and pathology of SARS-CoV-2	Molecular; tridimensional models	Integrins as cell receptors of SARS-CoV-2 in one or more host species, through a conserved RGD (403–405: Arg-Gly-Asp) motif present in the receptor-binding domain of the spike proteins of all SARS-CoV-2 sequences	Review	[58]
	Molecular	Nongenomic actions of T4 (tumor, endothelial cells) mediated by the binding of the extracellular domain of plasma membrane integrin ανβ3	Review	[59]
The state of immune activation accompanying inflammatory thyroid disease is comparable to the cytokine storm associated with COVID-19	Histological; cellular; molecular	Eight-week-old female CBA/J mice (a strain susceptible to experimental autoimmune thyroiditis) were immunized with thyroglobulin and then injected with IFN-γ and TNF-α vs. control animals.	In vivo study	[60]
	Molecular	IL-18^−/−^, IFN-γ^−/−^, and WT mice injected with bacterial lipopolysaccharide	In vivo study	[61]
	Cellular; molecular	Cytokines are implicated in the pathogenesis of autoimmune thyroid diseases, while cytokine modulation is a possible therapeutic target	Review	[62]
	Epidemiological, molecular	Cytokines activated during the inflammatory response are causally associated with the pathogenesis of NTIS, making NTIS part of the acute phase response	Review	[63]
Inverse correlation between serum levels of TSH and inflammatory cytokines in patients with COVID-19, which may explain NTIS or overt thyrotoxicosis	Epidemiological; clinical; molecular	Italy; 287 noncritically ill patients (193 men, median age 66 years	Retrospective single-center study	[41]
	Epidemiological; imaging; molecular	Italy; 144 consecutive patients (97 men, and 47 women, mean age 68.1 years) admitted to HICU or LICU	Prospective cohort study	[64]
	Epidemiological; clinical; molecular	Denmark; 116 consecutive patients hospitalized for moderate-to-severe COVID-19 disease	Retrospective single-center study	[65]
Relapse of GD described in COVID-19 patients	Clinical; imaging; molecular	A 60-year-old woman with a previous diagnosis of GD at the age of 23 years; A 53-year-old woman (no previous known thyroid disease)	Case reports	[66]
	Clinical; imaging; molecular	A 45-year-old woman with a 12-year medical history of GD; A 61-year-old woman with a history of atrial fibrillation and GD	Case reports	[67]

Abbreviations: ACE2—angiotensin-converting enzyme 2; AMI—acute myocardial infarction; CRF—chronic renal failure; EBV—Epstein–Barr virus; FT3—free triiodothyronine; GD—Graves’ disease; HFV—human foamy virus; HICU—high-intensity care unit; HIV—human immunodeficiency virus; HSV—herpes virus simplex; HTLV-1—human T-cell lymphotropic virus type 1; ICU—intensity care unit; IFN-γ—interferon gamma; IL—interleukin; LICU—low-intensity care unit; NTIS—non-thyroidal illness syndrome; SAT—subacute thyroiditis; SARS-CoV—severe acute respiratory syndrome coronavirus; SV40—Simian virus 40;_T3—triiodothyronine; TMPRSS2—transmembrane protease serine 2; TNF-α—tumor necrosis factor-alpha; TSH—thyroid-stimulating hormone; WT—wild type.

**Table 2:** Summary of potential pitfalls in the relationship between COVID-19 and thyroid dysfunction.

Pitfalls	Reference
No possibility to infer a causal relationship and to investigate the potential impact of thyroid dysfunction on COVID-19 outcomes due to: - retrospective design - cross-sectional design - single-center design - small sample size - association mainly relied on case reports	[41,50,51,65] [68] [41,51,52,64,65,68] [41,50,51,65,68] [30,31,32,33,34,35,36,37,38,39,40]
Differences: -in the studied populations (critically ill vs. noncritically ill subjects) - in the timing of thyroid function assessment with respect to the course of the disease	[41,47,48,49,50,51,52,65,68] [30,31,32,33,34,35,36,37,38,39,40,47,48,49,50,51,65,68]
Lack of measurement of: - thyroid hormones - thyroglobulin - autoantibodies	[47,52,65] [41,47,50,51,52,65] [47,50,51,52,65]
The potential confounding effect of medications used in COVID-19 (glucocorticoids, low-molecular-weight heparin) due to their impact on the HPT axis and free thyroid hormone assays	[41,50,51,69]
Difficult generalization of results due to lack of a control group of healthy individuals or an independent cohort of patients with non-COVID-19 pneumonia	[41,48,64,65]
SARS-CoV-2 was not directly detected in the thyroid tissue in all case studies of SAT	[30,31,32,33,34,35,36,37,38,39,40]
Differently from what happens in infection with SARS-CoV: no significant damage to thyroid cell morphology - rare development of hypothyroidism in COVID-19 patients	[41] [41,47,48,49,66,70]
The association between thyroid function and COVID-19 most studied in nonmild cases	[47,50,51,52,64,65,69]
Hypothyroidism is not associated with complications of COVID-19	[71]
Possibility of publication bias in the relationship between COVID-19 and thyroid dysfunction	[72]

Abbreviations: HPT—hypothalamic-pituitary-thyroid; SARS-CoV—severe acute respiratory syndrome coronavirus; SAT—subacute thyroiditis.

**Table 3:** Key selenoproteins relevant for thyroid pathophysiology.

Family	Acronym	Enzymes	Main Functions	Reference
Iodothyronine deiodinases	DIO	DIO1, DIO2, DIO3	Thyroid hormone activation/inactivation (T4 in T3 conversion; T4 in rT3 and T3 in T2 conversion)	[146]
Glutathione peroxidases	GPx	GPx1, GPx2, Gpx3, GPx4	Free radical scavenger Protection against inflammation H_2_O_2_ reduction and prevention of lipid peroxidation Maintenance of intracellular homeostasis and redox balance	[147]
Thioredoxin reductases	TrxR	TrxR1, TrxR2	NAPH-dependent oxidoreductase activity Regulation of cell proliferation (apoptosis)	[148,149]

Abbreviations: H_2_O_2_—hydrogen peroxide; rT3—reverse triiodothyronine; T2—3,5-diiodothyronine; T3—triiodothyronine; T4—thyroxine.

## Data Availability

Not applicable.

## Abbreviations

ACE2	Angiotensin-converting enzyme 2
CRP	C-reactive protein
DIO	Deiodinase
FT3	Free triiodothyronine
FT4	Free thyroxine
GD	Graves’ disease
GPx	Glutathione peroxidase
ICU	Intensive care unit
IFN-γ	Interferon-gamma
IL	Interleukin
NF-kB	Nuclear factor-kB
NTIS	Non-thyroidal illness syndrome
SARS-CoV-2	Severe acute respiratory syndrome coronavirus 2
SAT	Subacute thyroiditis
Se	Selenium
Sec	Selenocysteine
SELENOP	Selenoprotein P
SeNPs	Selenium nanoparticles
T3	Triiodothyronine
T4	Thyroxine
TNF-α	Tumor necrosis factor-alpha
TrxR	Thioredoxin reductases
TSH	Thyroid-stimulating hormone
Zn	Zinc
